# The effects of Ramadan fasting on the number of renal colic visits to the emergency department

**DOI:** 10.12669/pjms.321.8248

**Published:** 2016

**Authors:** Yunsur Cevik, Seref Kerem Corbacioglu, Gulsah Cikrikci, Veysel Oncul, Emine Emektar

**Affiliations:** 1 Dr. Yunsur Cevik, Associate Professor of Emergency Medicine, Kecioren Training and Research Hospital, Emergency Medicine Department, Ankara / Turkey; 2 Dr. Seref Kerem Corbacioglu Emergency Medicine Specialist, Kecioren Training and Research Hospital, Emergency Medicine Department, Ankara / Turkey; 3 Dr. Gulsah Cikrikci, Emergency Medicine Resident, Kecioren Training and Research Hospital, Emergency Medicine Department, Ankara / Turkey; 4 Dr. Veysel Oncul, Emergency Medicine Resident, Kecioren Training and Research Hospital, Emergency Medicine Department, Ankara / Turkey; 5 Dr. Emine Emektar, Associate Professor of Emergency Medicine, Kecioren Training and Research Hospital, Emergency Medicine Department, Ankara / Turkey

**Keywords:** Emergency Medicine, Fasting, Ramadan, Renal colic

## Abstract

**Objective:**

The effects of fluid and diet restriction strictly during the long hours in Ramadan on the number of colic visits and biochemical factors of stone formation are controversial in the literature. The aim of this study was to assess the effects of Ramadan fasting on the number of renal colic visits and laboratory results of patients with renal colic.

**Methods:**

This was a prospective observational study, which was conducted with patients who were admitted to our emergency department with renal colic. The study period was divided into two parts: Before Ramadan and Ramadan. All laboratory results of patients and daily air temperature values were recorded. p<0.05 was considered statistically significant for all tests.

**Results:**

Total 176 patients (n:89 in before Ramadan, n:87 in Ramadan) with renal colic were enrolled into the study. During Ramadan, 49 (73.1%) of 67 patients were admitted in the first half of the month and 20 patients (26.9%) were admitted in the second half of the month. Only urine density and white blood cell values in Ramadan and non-Ramadan period were significantly different (p=0.004 and p=0.001). Hemoglobin, general crystal, and triple phosphate crystal values in the first and the second half of Ramadan were significantly different (p=0.04, p=0.03, and p=0.03).

**Conclusion:**

This study has shown that fasting in Ramadan does not change the number of renal colic visits. In addition, although fasting causes some changes in urinary metabolites, there is not enough evidence that these changes increase urinary calculus formation.

## INTRODUCTION

Renal colic, which is known as kidney stones, is a common disorder in the emergency departments (ED) and accounts for approximately 0.6% of ED visits.^1^ Previous studies have shown that the incidence of renal stone disease changes seasonally.^2^ Especially, the highest incidence rates have been reported during the warmer months such as July, August, and September.^2^,^3^ Possible reasons of increased incidence have been described in the literature. One of these reasons is dehydration due to perspiration, which is common on warmer months when patients persistently have low urine volume and high concentration urine contains which can increase the risk of stone formation.^4^ The other reason is increased urine calcium excretion during warmer months.^5^ In addition to low fluid intake as a risk factor for stone disease, increasing fluid intake can cause a reduction in recurrent stone formation.^6^

Ramadan is the ninth month of the lunar year (Hijri) and Muslims abstain from eating and drinking from sunrise to sunset during Ramadan. As Hijri year is 11 days shorter than the solar year, Ramadan rotates around all four seasons once every 33 years. Thus, daily fasting period can last 15-16 hours on summer. Fluid and diet restrictions during the month long intermittent Ramadan fast can influence the biochemical factors related to stone formation. Yet, studies on the effects of Ramadan fasting on the incidence of renal colic are scarce and have given variable and inconclusive results.

The primary aim of this study was to assess the effects of Ramadan fast and temperature changes on the number of renal colic visits to the local emergency department. The second aim of this study was to determine whether there was a difference in urine analyses results and biochemical parameters during and after Ramadan.

## METHODS

This prospective observational study was conducted in an emergency department (ED) of a Training and Research Hospital between May 28 and July 27, 2014 during 2-month period. The study period was divided into two parts: a month before Ramadan (non-Ramadan period) and during the month of Ramadan. The temperature was recorded every day during the study. The Local Ethics Committee approved the study protocol. Written informed consent was obtained from all patients.

The inclusion criteria included: patients who were admitted to our ED; age 18 years or older with renal colic; and the absence of any exclusion criteria. The exclusion criteria included: age younger than 18 years; patients who refused to be involved in the study; patients whose renal colic diagnoses were unclear, patients with co-morbid diseases such as chronic kidney disease, metabolic disorder, cardiovascular disease, liver or endocrine disorder. In addition, when urine analyses results and biochemical parameters of Ramadan and non-Ramadan period were compared, patients who were not fasting every day during Ramadan were excluded.

The diagnosis of renal colic was made based on physician’s clinical judgment according to classical clinical features, history, and physical examination. Sudden onset of colicky pain begins in the flank that radiates to the groin with hematuria, nausea, vomiting, dysuria, and urgency are clinical features. Complete blood count, biochemical tests, and spot urine test were performed for all patients. Non-contrast abdominal computed tomography (CT) was performed for patients who had no definite diagnoses of renal colic performed and after CT, patients who were diagnosed with other than renal colic were excluded.

All statistical calculations were performed using SPSS statistical software (version 15.0, SPSS Inc., Chicago, IL, USA). The Shapiro-Wilk test was used in order to evaluate whether demographic data, temperature values, blood, and urine results of the patients were normally distributed. Mann-Whitney U test was used in order to compare blood and urine results of patients who were admitted on different periods. p<0.05 was considered statistically significant for all tests.

## RESULTS

Total 176 patients (89 patients in a month non-Ramadan and 87 patients during Ramadan) with clinically diagnosed renal colic were enrolled into the study. The mean age was 40.47±12.32 years (range 18-81 years). There were 112 males (63.6%) and 64 females (36.4%). The mean air temperature of non-Ramadan and Ramadan month was 24.26±3.55 (18-33)°C and 30.76±2.42 (26-35)°C respectively. The difference between the mean temperature of non-Ramadan and Ramadan months was statistically significant (p<0.001). Although the mean air temperature of Ramadan month was higher than the mean air temperature of Non-Ramadan month, it was found that this difference did not increase the numbers of renal colic visit.

In non-Ramadan period, the mean age of the patients was 36.57±10.57 years and 61 (68.5%) of 89 patients were male. 20 of 87 patients who were admitted in the month of Ramadan were excluded because of non-fasting. The mean age of the rest (67 patients) who were included into the study and were fasting during Ramadan period were 44.75±12.50 years. 44 (65.7%) of 67 patients were male. During Ramadan, 49 (73.1%) of 67 patients were admitted in the first half of the month and 20 patients (26.9%) were admitted in the second half of the month. Urine and blood analyses of subjects revealed that only urine density and white blood cell values in Ramadan and non-Ramadan period were significantly different (p=0.004 and p=0.001) (Table-I). When blood and urine analyses results of patients in the first and the second half of Ramadan month were compared, significant differences were found in hemoglobin, general crystal, and triple phosphate crystal values (p=0.04, p=0.03, and p=0.03) (Table-II).

**Table-I:** Demographic and laboratory analysis of patients admitted at Ramadan and Non-Ramadan Periods.

	Non-Ramadan	Ramadan	P value
Patients Age	36.57±10.57	44.75±12.50	<0.001
Temperature (C^0^)	24.26±3.55	30.76±2.42	<0.001
WBC count (10^3^/mm^3^)	10,6±3,0	8,9±2,3	0.001
Hemoglobine (g/dL)	14.22±1.6	14.38±1.5	0.7
Urea (mg/dL)	31.26±8.83	33.85±7.56	0.055
Creatinine (mg/dL)	1.08±0.25	1.06±0.20	0.78
Sodium (mmol/L)	138.38±2.77	139.42±2.62	0.17
Potassium (mmol/L)	4.06±0.38	4.14±0.30	0.15
Calcium (mg/dL)	9.55±0.43	9.56±0.35	0.89
Urine density (Specific gravity)	1020.79±9.23	1024.93±7.86	0.004
Urine ketone (mg/dL)	0.39±1.83	0.17±0.87	0.39
Urine leukocyte (p/HPF)	16.34±52.04	7.15±21.90	0.34
Urine erythrocyte (p/HPF)	60.29±101.77	79.37±115.03	0.25
Renal epithelia (p/HPF)	0.71±2.81	0.59±1.54	0.17
Amorphous crystal (p/HPF)	5.10±16.01	3.34±19.20	0.04
General crystal (p/HPF)	6.10±16.50	6.45±22.85	0.8
Calcium oxalate (p/HPF)	0.27±1.37	1.67±9.34	0.053
Triple phosphate crystal (p/HPF)	0.04±0.24	0.01±0.06	0.9

WBC: White blood cell HPF: High-power Field. Values are expressed mean ± standard deviation (SD),

**Table-II:** Demographic and laboratory analysis of patients admitted at different periods of Ramadan Months.

	First Half of Ramadan	Second Half of Ramadan	P value
Patients Age	42.38±12.36	51±10	0.007
Temperature (C^0^)	30.69±2.8	30.94±0.2	0.4
WBC count (10^3^/mm^3^)	8944±2444	9144±1975	0.7
Hemoglobine (g/dL)	14.6±1.4	13.7±1.9	0.04
Urea (mg/dL)	34.16±6.9	33±9.1	0.2
Creatinine (mg/dL)	1.07±0.18	1.03±0.22	0.4
Sodium (mmol/L)	139±2.9	139±1.3	0.5
Potassium (mmol/L)	4.13±0.3	4.13±0.1	0.4
Calcium (mg/dL)	9.5±0.3	9.4±0.3	0.2
Urine density (Specific gravity)	1024±8.5	1027±4.9	0.4
Urine ketone (mg/dL)	0.2±0.9	0.08±0.35	0.8
Urine leukocyte (p/HPF)	8.9±25	2.2±2.6	0.7
Urine erythrocyte (p/HPF)	68±101	109±144	0.1
Renal epithelia (p/HPF)	0.7±1.7	0.27±0.69	0.1
Amorphous crystal (p/HPF)	4.5±22	0.0±0.0	0.3
General crystal (p/HPF)	7.4±26	3.6±7.9	0.03
Calcium oxalate(p/HPF)	2±10	0.5±1.1	0.3
Triple phosphate crystal(p/HPF)	0.009±0.06	0.03±0.08	0.03

WBC: White blood cell HPF: High-Power Field. Values are expressed mean ± standard deviation (SD).

## DISCUSSION

Actually, expected numbers of renal colic visit in the month of Ramadan are more than non-Ramadan period because of fluid and diet restriction strictly during the long hours in Ramadan. However, previous studies have shown that the accuracy of this exception is controversial. Al-Hadramy et al. reported that highest rates of renal colic admission were found on June, July, and August in which the highest temperatures were as 30-32°C. Nevertheless, rates of renal colic admission in Ramadan were similar to rates of other months.^7^ Basiri et al.^3^ investigated the effect of Ramadan on the number of renal colic visits and they found that there was no significant difference between the frequencies of patients with stone colic in Ramadan and non-Ramadan months which air temperatures were similar. Similarly, Al-Hadramy et al. have reported that the highest frequencies of patients with stone colic were in warmer months.^7^ Contrary to these studies, Abdulreza et al reported that numbers of admission with renal colic (n: 195) to the ED during the first half of Ramadan were significantly higher than numbers of two weeks (n: 157) before the month of Ramadan and numbers of two weeks (n: 119) after the month of Ramadan. However, numbers of admission during to second half of Ramadan (n: 139) were similar to other periods.^8^ Similar to first two studies, the results of our study showed that numbers of renal colic visit in the month of Ramadan were not significantly different from non-Ramadan period.

At the same time, similar to study of Abdulreza et al.,^8^ there was significant reduction in the numbers of renal colic visit in the second half of Ramadan compared to visit numbers of first half of Ramadan in our study. According to Abdulreza et al.^8^, the reason of this increase in hospital admissions at the beginning of Ramadan may be a sudden change in dietary habits (especially reduced water intake) and then this increase is normalized due to adaptive mechanisms of the body.

There are very few reports on urine and blood tests associated with Ramadan in the literature. Zghal et al.^9^ aimed to evaluate the effect of fluid and diet restriction in Ramadan on biochemical factors of stone formation. They divided 90 patients into three groups: healthy fasting patients (GI), healthy non-fasting patients (G2), and non-fasting patients with calcium lithiasis (G3). They reported that supersaturation of urine with oxalate, uric acid, and brushite were the same for (G1) and (G3) and higher than (G2). In addition, they reported that crystalluria was more important in lithiasis subjects compared with healthy non-fasting patients.^9^ In the study that was conducted by Miladipour et al.^10^ on effect of Ramadan fasting on urinary factors, 24-hour urine samples of 37 patients with recurrent calcium calculus formers and 20 volunteers with no history of kidney disease or calculus formation were analyzed before the beginning of Ramadan and during Ramadan. It was reported that total excretion of calcium, phosphate, and magnesium in 24-hour urine samples and also urine volumes during fasting were significantly lower than those in the non-fasting period. In addition, no significant increase in calcium oxalate supersaturation was reported during the fasting period.^10^ In our study we did not analyze 24-hour urine samples. We only analyzed spot urine test and we found significant difference in urine density between Ramadan and non-Ramadan period. In addition, we compared blood and urine analyses results of patients in the first half and the second half of Ramadan month and we found differences on some parameters such as hemoglobin, general crystal, and triple phosphate crystal values. However, in our opinion, although these differences are statistically significant, it is unclear how to use these differences in clinical practice.

### Limitations of the study

Our study has two important limitations. First and the most important limitation is temperature values of the month of Ramadan were significantly higher than values of non-Ramadan period. Because of this difference, we expected to encounter false increase in admission due to renal colic. However, admissions due to renal colic were similar in both periods. Therefore, we think that this limitation is not significant. The second limitation is that we could not perform abdominal computed tomography for all patients because of potential cancer risk and ethical concerns. Therefore, diagnosis of renal colic was not made by gold standard test and this decision was based more clinical judgment.

## CONCLUSION

This study has shown that fasting in Ramadan does not seem a factor that is the reason of increase in the number of renal colic visits. Fasting causes some changes in urinary metabolites that have different effects on calculus formation. However, there is not enough evidence that these changes increase urinary calculus formation and it is unclear how to use these changes in our clinical practice. The effect of fasting on renal colic incidence, urinary factors, and biochemical parameters needs to be investigated by further studies.
